# Quercetin activates vitamin D receptor and ameliorates breast cancer induced hepatic inflammation and fibrosis

**DOI:** 10.3389/fnut.2023.1158633

**Published:** 2023-04-20

**Authors:** Nirmala G. Sannappa Gowda, Varsha D. Shiragannavar, Lakshana D. Puttahanumantharayappa, Ashwini Tumkur Shivakumar, Siva Dallavalasa, Chaithanya G. Basavaraju, Smitha S. Bhat, Shashanka K. Prasad, Ravishankar M. Vamadevaiah, SubbaRao V. Madhunapantula, Prasanna K. Santhekadur

**Affiliations:** ^1^Department of Biochemistry, Center of Excellence in Molecular Biology and Regenerative Medicine, JSS Medical College, JSS Academy of Higher Education and Research, Mysore, India; ^2^Department of Conservative Dentistry and Endodontics, JSS Dental College and Hospital, Mysore, Karnataka, India; ^3^Department of Biotechnology and Bioinformatics, JSS Academy of Higher Education and Research, Mysore, Karnataka, India; ^4^Bioactive Compound Laboratory, Faculty of Agriculture, Chiang Mai University, Chiang Mai, Thailand; ^5^Department of Anatomy, JSS Medical College, AHER, Mysore, Karnataka, India

**Keywords:** Ehrlich ascites carcinoma, quercetin, vitamin D3, vitamin D receptor, hepatic inflammation

## Abstract

**Aims:**

To explore the hepatoprotective role of quercetin and its novel molecular mechanism of action on breast cancer associated hepatic inflammation and fibrosis *via* Vitamin D receptor (VDR).

**Main methods:**

We used Ehrlich Ascites Carcinoma (mouse mammary carcinoma) model for our *in-vivo* experiments and human breast cancer cell lines for *in-vitro* assays. We inoculated 1.5 × 10^6^ Ehrlich ascites carcinoma cells into female Swiss albino mice. Quercetin (50 mg/kg) was administered intraperitoneally for 15 days. Liver enzymes activity was determined using a spectrophotometric assay. The hallmarks of inflammation and fibrosis were determined using Immunohistochemistry. The effect of quercetin on tumor formation was elucidated using human breast cancer cell lines and chick chorioallantoic membrane assay. Docking study was performed to explore the binding mode of quercetin with VDR.

**Key findings:**

In EAC tumor-bearing mice, cell numbers, tumor volume, body weight and liver weight were dramatically increased, while they significantly decreased in mice treated with quercetin. Additionally, the peritoneal neo-angiogenesis was also significantly suppressed in the quercetin-treated mice, compared to the control. In addition, quercetin treated EAC tumor bearing mice had lower levels of liver enzymes, decreased hepatic inflammation and fibrosis compared with EAC tumor bearing mice. Docking study confirmed VDR-quercetin interaction. Furthermore, *in-vitro* assays and chick chorioallantoic membrane assay revealed the Vitamin D mimicking effect of quercetin.

**Significance:**

Dietary flavonoid, quercetin could act as a promising therapeutic drug to suppress the breast cancer induced tumor angiogenesis, hepatic inflammation, and fibrosis possibly *via* activation of VDR.

## 1. Introduction

Breast cancer is one of the major and the most known and reported malignancies among women population around the world, and it is also the leading and major cause of cancer-related deaths in women. Breast cancer has the highest prevalence and mortality rate in India and the world according to the 2020 Globocan cancer report (1). Age, family history, mutation, lifestyle, obesity, and other factors play a significant role in the development of breast cancer (2). Aside from these risk factors, the mortality rate of breast cancer patients has been linked to a wide range of other diseases (3). Thus, it is critical to understand the molecular relationship between breast cancer and other related comorbidities. Some clinical studies suggest that, patients with breast cancer shows most prevalent comorbidities such as hypertension, arthritis, thyroid problem, hypercholesterolemia and diabetes (4). Breast cancer is also directly associated with obesity and Nonalcoholic Fatty liver disease (5, 6). There are many reports which shows that liver metastasis is very common in breast cancer patients (7, 8). Our own previous study showed that Breast cancer associated mice model can be used to study the hepatic inflammation and fibrosis (9). Based on that, here we aimed to further understand the molecular mechanism between breast cancer induced liver inflammation and fibrosis and also it can be adopted to the development of therapeutic approaches to treat breast cancer induced liver inflammation and fibrosis.

The liver is a vital organ involved in regulating metabolic homeostasis and it has a unique vascular supply and it receives most of the blood from the portal venous circulation. Therefore, any pathological change in liver functional and architectural pattern may leads to various diseases (10). Angiogenesis is the biological process of establishment of new blood vessels from pre-existing one, and is reported in several pathophysiological conditions (11). Abnormal or defective liver angioarchitecture is directly linked to hepatitis, which may lead to cirrhosis and cancer (12). Tumor angiogenesis is a vital process that provides a supportive nutrients and oxygen rich microenvironment to the growing tumor and may also play a significant role in the development of hepatitis through multiple signaling pathways (13). Various findings also suggest that tumor angiogenesis as one of the distinguishing signatures for the development of liver inflammation and fibrosis, which eventually leads to hepatocellular carcinoma (HCC) (14, 15). As a result, inhibition of tumor-angiogenesis will be a favorable therapeutic approach to prevent the breast cancer induced liver injury (16). Currently, dietary natural compounds are gaining popularity due to their limited or no side effects nature (17). Therefore, we attempted to explore the beneficial effects of a dietary natural compound that ameliorates breast cancer induced liver injury *via* novel signaling pathways (9).

Quercetin is a dietary flavonoid, which is abundant in various fruits and vegetables and possess anti-inflammatory, anti-tumor, anti-oxidants and other beneficial effects (18). Recent reports have shown that quercetin inhibits various cancer hallmarks and oncogenic signaling pathways in breast cancer cells (19, 20). However, the potential effect of quercetin and its molecular mechanism in inhibiting breast cancer mediated hepatitis is not studied and needs to be investigated.

To explore the potential beneficial effects of quercetin, we examined its effect on EAC mice model as well as on human breast cancer cell lines. Since the receptor for vitamin D are ubiquitously present in all cells, vitamin D3 acts as a ligand for the activation of Vitamin D receptor (21). Although few reports have also shown that quercetin directly interacts with vitamin D receptor, but the molecular mechanism is poorly understood and requires experimental validation (22, 23). Here, we tried to explore the possible link between tumor angiogenesis and liver injury and novel possible hepatoprotective effect of quercetin and its vitamin D mimicking action *via* VDR in breast cancer.

## 2. Materials and methods

### 2.1. Cells and materials

EAC (Ehrlich ascites carcinoma cells) and human Breast cancer cell lines (MDA-MB-231 and BT-474) were kind gift from Dr. MVVST SubbaRao, CEMR laboratory. Quercetin (Q4951) and vitamin D3 (C9756) were procured from Sigma-Aldrich, United States. WST-1 reagent was procured from TAKARA. Fertilized chicken eggs were procured from Ilavala poultry farm, Mysuru. The Ehrlich ascites carcinoma (EAC) model is very commonly used model to study the pathological conditions of breast cancer and associated tumor angiogenesis. These EAC cells were first discovered and isolated by the Nobel Laureate, Paul Ehrlich in the mammary gland tumor of a white mouse, therefore these tumor cells were named after him to honor his great contribution to tumor biology. Recently, we further developed this model to study the breast cancer induced liver inflammation and fibrosis (9).

### 2.2. Animal care and handling

The Institutional Animal Ethics Committee (JSSAHER/CPT/IAEC/087/2021), JSS Medical College, JSS AHER, Mysore, Karnataka, India approved the animal experimental procedure. A total of 15 female Swiss albino mice were procured from M/s. Adita Biosys, Tumakuru, which were weighing 18–21grams and aged between 5 and 6 weeks and then housed for 7 days prior to all *in-vivo* studies. All the animals were kept in polypropylene cages at a constant room temperature of 25°C ± 3°C, with a relative humidity of 45%–55% and a 12 h light/12 h dark cycle (artificial photoperiod). The animals were fed with a rodent normal chow diet and mineral water on an *ad libitum* basis.

### 2.3. Inoculation of tumor cells

1.5 × 10^6^ EAC cells were injected into a donor mouse’s peritoneal cavity. After 10 days, ascites fluid containing EAC cells was collected from donor mice and the cells were counted using a hemocytometer. After counting, 100 μL of fluid was administered into each recipient mouse’s peritoneal cavity, which contained 1.5 million viable cells (24).

### 2.4. Experimental design

**Figure 1:**
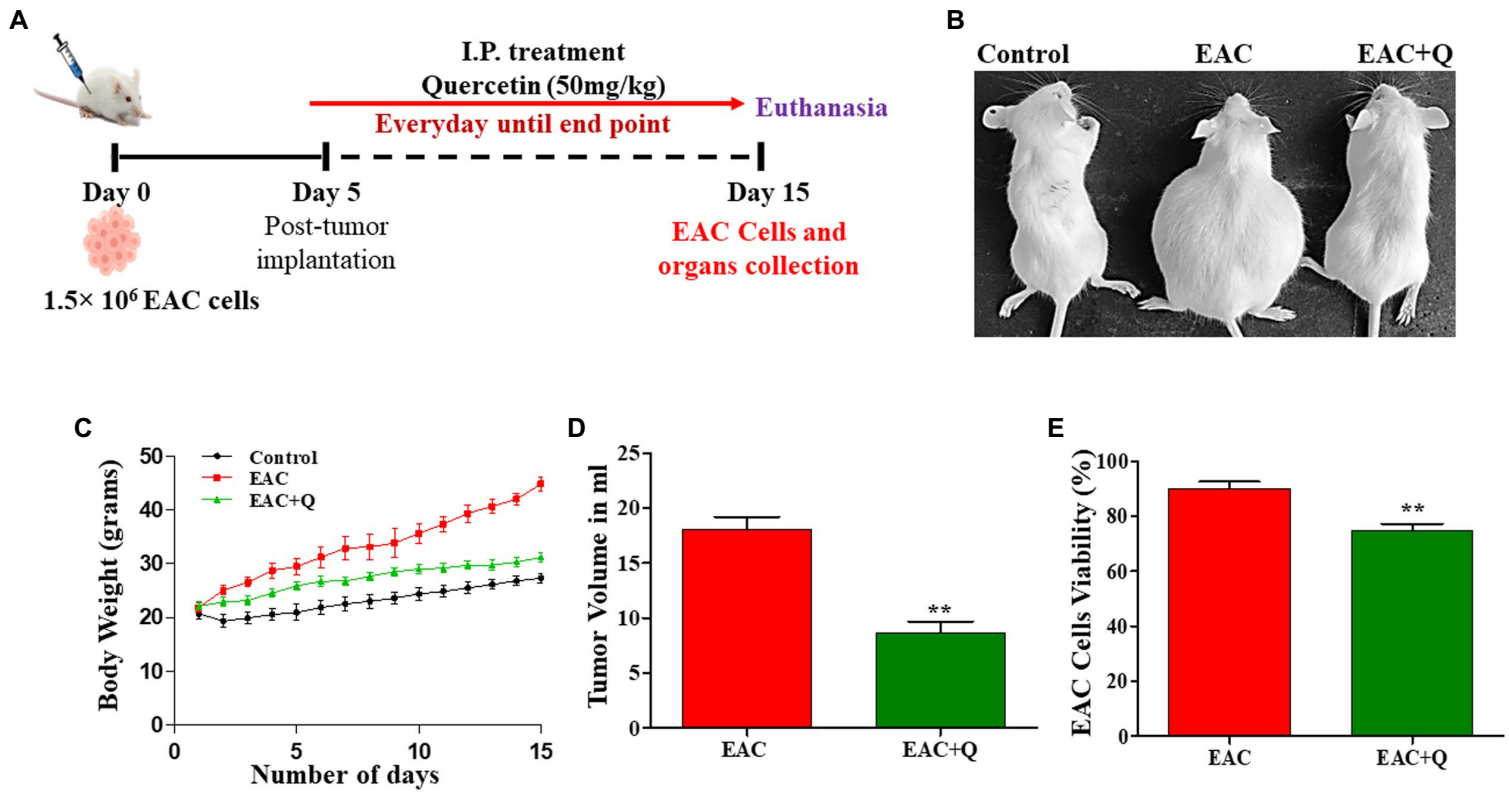
Quercetin has an anti-tumor effect against EAC cells. **(A)** Schematic treatment pattern of quercetin-EAC liquid tumor model. **(B)** Image representing the reduction of body weight in quercetin treated EAC mice compare to control EAC bearing mice. **(C)** Graph representing the decrease in the body weight of quercetin treated EAC animals compare to control EAC bearing animals (*n* = 5/group). **(D,E)** Effect of quercetin on tumor volume and EAC cell viability (%) when compared to control EAC bearing animals (*n* = 5/group). ** represents that *p* values less than 0.005.

After 3–4 days of post tumor inoculation, the inoculated mice were separated into three distinct groups (5 mice per cage) according to the treatment pattern as follows: The non-tumor bearing mice were considered as the negative control group. The EAC group containing EAC tumor cells considered as the positive control group. The remaining EAC group was treated with quercetin every day until the 15th day (Figure 1A). The quercetin dose was prepared using 0.01% DMSO, and each mice received 50 mg/kg intra-peritoneally (25). All animals were anaesthetized on the 15th day, blood was collected by retro orbital (processed serum sample were stored at −80°C), and then animals were sacrificed by cervical dislocation. Each group’s peritoneal ascites fluid was collected for measurements of tumor proliferation and progression parameters (volume of ascites fluid and cell viability), as well as the liver and peritoneal cavity layer was collected and immediately fixed in 10% formaldehyde and used for further experiments.

### 2.5. Biochemical estimation

Elevated liver enzymes indicate the hepatic inflammation or hepatocyte damage. Mouse liver enzymes [Alanine Transaminase (ALT), Aspartate Transaminase (AST) and Alkaline Phosphatase (ALP)] activity was determined by adding an enzyme specific working reagent to each serum sample and incubating at 37°C for 1 min, and measuring the change in absorbance per minute during 3 min using a semi-autoanalyzer (MISPA VIVA) according to the manufacturer’s instructions (Agappe diagnostics Ltd., India).

### 2.6. Histopathological estimation

The liver tissue and peritoneum of each mouse was harvested and instantly fixed in neutral buffered formalin (10% formaldehyde) and embedded in paraffin after dehydration of ethyl alcohol series for further processing. Five micrometer thick sections of liver tissue were stained with Hematoxylin and eosin staining (H&E) and Trichome Masson staining (TMS) (9). The intensity of inflammation was determined using H&E staining, and the degree of fibrosis was determined using TMS. To measure the degree of fibrosis, we used the scoring pattern based on the intensity of staining ranging from 0 (no fibrosis) to 5 (severe fibrosis). Five micrometer thick sections of peritoneum were subjected to immunohistochemistry to visualize the expression of CD31 marker as well as inflammation level by H&E staining (26).

### 2.7. Cell proliferation assay

To measure the rate of cell proliferation of human breast cancer cell lines, the Water-soluble Tetrazolium-1 (WST-1) cell proliferation assay was performed. Cells were cultured in DMEM medium with 10% FBS and 100 U/mL streptomycin at 37°C and 5% CO_2_ under humidified atmospheric conditions. 10,000 cells per well were plated in 96 well plates and treated for 24 h with various concentrations of quercetin (20–100 μM), vitamin D3 (6.25–200 μM), and a combination of both. After 24 h, 10 μL premixed WST-1 reagent was added to each experimental well, and the plates were incubated for 3 h at 37°C in 5% CO_2_. After incubation, absorbance was measured at 450 nm using a PerkinElmer multimode plate reader. The results were represented in percentage of inhibition by using the formula: % of Inhibition = Test/Net × 100 and the combination index (CI) was calculated by using the multiple drug-effect equation of Chou-Talalay in the CompiSyn program. Combination index (CI) represent the type of interaction between two administered drugs. The Chou-Talalay method for drug combination is based on the median-effect equation, which provides the theoretical basis for the combination index (CI)-isobologram equation that allows quantitative determination of drug interactions, where CI < 1 indicates synergism, CI = 1 indicates additive effect and CI > 1 indicates antagonism, respectively.

### 2.8. Chick chorioallantoic membrane assay

The angiogenic activity was performed by using chick embryos as described by Prasanna et al. (27). A Chick chorioallantoic membrane (CAM) assay was done to explore the effects of quercetin and vitamin D3 on *ex-vivo* angiogenesis. Fertilized eggs were incubated at 37°C until the 8th day. On the 9th day, a small window was made on the top of the live eggs shell by using a sterile blade. Quercetin and vitamin D3 stocks were prepared by dissolving in DMSO followed by diluted with PBS to get desired concentration and gently applied on the CAM. The eggs were then photographed after a 48-h of incubation period.

### 2.9. Molecular docking

Auto Dock Vina was used to evaluate the binding affinity of quercetin with the vitamin D receptor (28). The docking investigation used the crystal structure of the VDR complexed with quercetin as the target structure. For the docking study, Water molecules were eliminated and further checked for any prior attachment to ligands and removed from the dimensional structure with the aid of version 2.4 of the PYMOL tool. To determine the binding affinity of quercetin as a ligand with the vitamin D receptor, docking poses and score calculations were performed.

### 2.10. Data analysis

Statistical analysis for all these experimental results were performed by using standard statistical software (Graphpad Prism version 5.0). All data were represented as means ± Standard Deviation. One-way ANOVA followed by Bonferroni’s Multiple Comparison Test was used to test the statistically significant difference among groups where * represents that *p* values less than 0.05. The staining images were captured from stereo zoom microscope with a CCD Olympus camera attached to it (cellSens Dimension 1.12). The cell proliferation images were captured from Carl Zeiss microscope. All the data is shown in *n* = 5.

## 3. Results

### 3.1. Quercetin has an anti-tumor effect against EAC cells

Quercetin-treated EAC animals showed significantly decreased (*p* < 0.0001) body weight when compared to control EAC-bearing animals (Figures 1B,C). To assess the anti-tumor effect of quercetin against EAC cells, changes in tumor volume and EAC cell viability were measured. Quercetin-treated EAC animals showed a significant reduction in tumor volume (*p* < 0.0012) on the 15th day of tumor inoculation when compare to control EAC bearing animals (Figure 1D). Also, quercetin-treated EAC animals showed a slight decrease in EAC cell viability (*p* < 0.0063) in comparison to EAC bearing animals (Figure 1E). Further, we found interesting changes in liver structure and we measured the liver weight and liver enzyme activity to assess the hepatoprotective role of quercetin on EAC mice.

### 3.2. Quercetin ameliorates EAC tumor cells induced hepatitis

**Figure 2:**
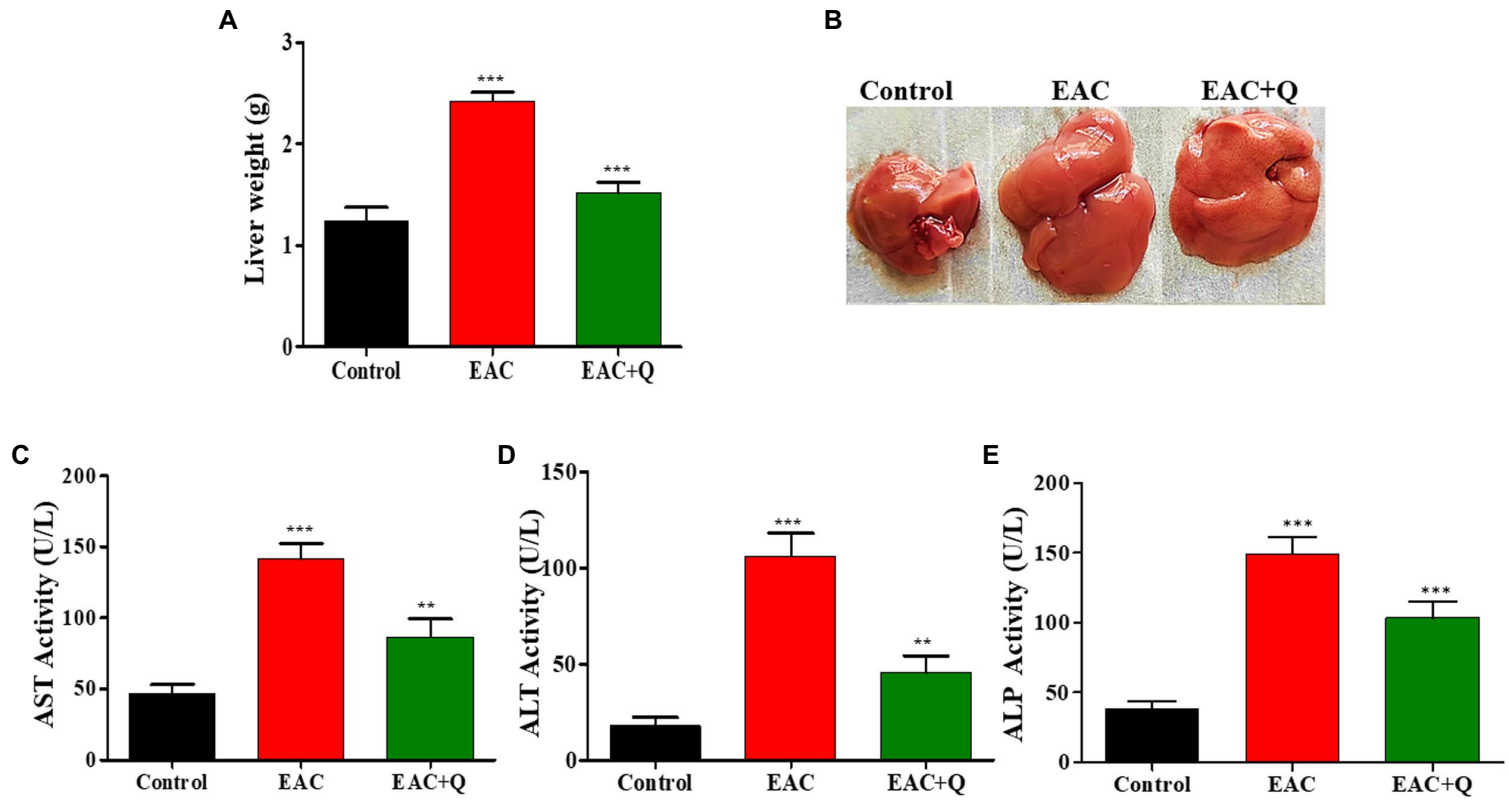
Quercetin ameliorates EAC tumor cells induced hepatitis. **(A)** Bar graph representing the decrease in liver weight of quercetin treated EAC mice compare to control EAC bearing mice (*n* = 5/group). **(B)** Representative images showing the decreased liver weight in quercetin treatment group (*n* = 5/group). **(C–E)** Bar graph showing the decrease in the serum AST, ALT, and ALP levels in quercetin treated EAC animals when compared to control EAC bearing animals (*n* = 5/group). **, *** represents that *p* values less than 0.005 and 0.0005 respectively.

Biochemical and physiological changes in liver functions are major hallmarks that reveals the gradual progression of liver injury (29). When we observed the liver weight, quercetin-treated EAC animals showed dramatic reduction (*p* < 0.0001) in liver weight when compared to control EAC bearing animals (Figures 2A,B) Biochemical assay results showed a significant decrease in the enzymes ALP (*p* < 0.0002), AST (*p* < 0.0001), and ALT (*p* < 0.0001) in quercetin-treated EAC animals compared to control EAC bearing animals (Figures 2C–E).

### 3.3. Quercetin suppresses the peritoneal angiogenesis and angiogenic marker, CD31 expression

**Figure 3:**
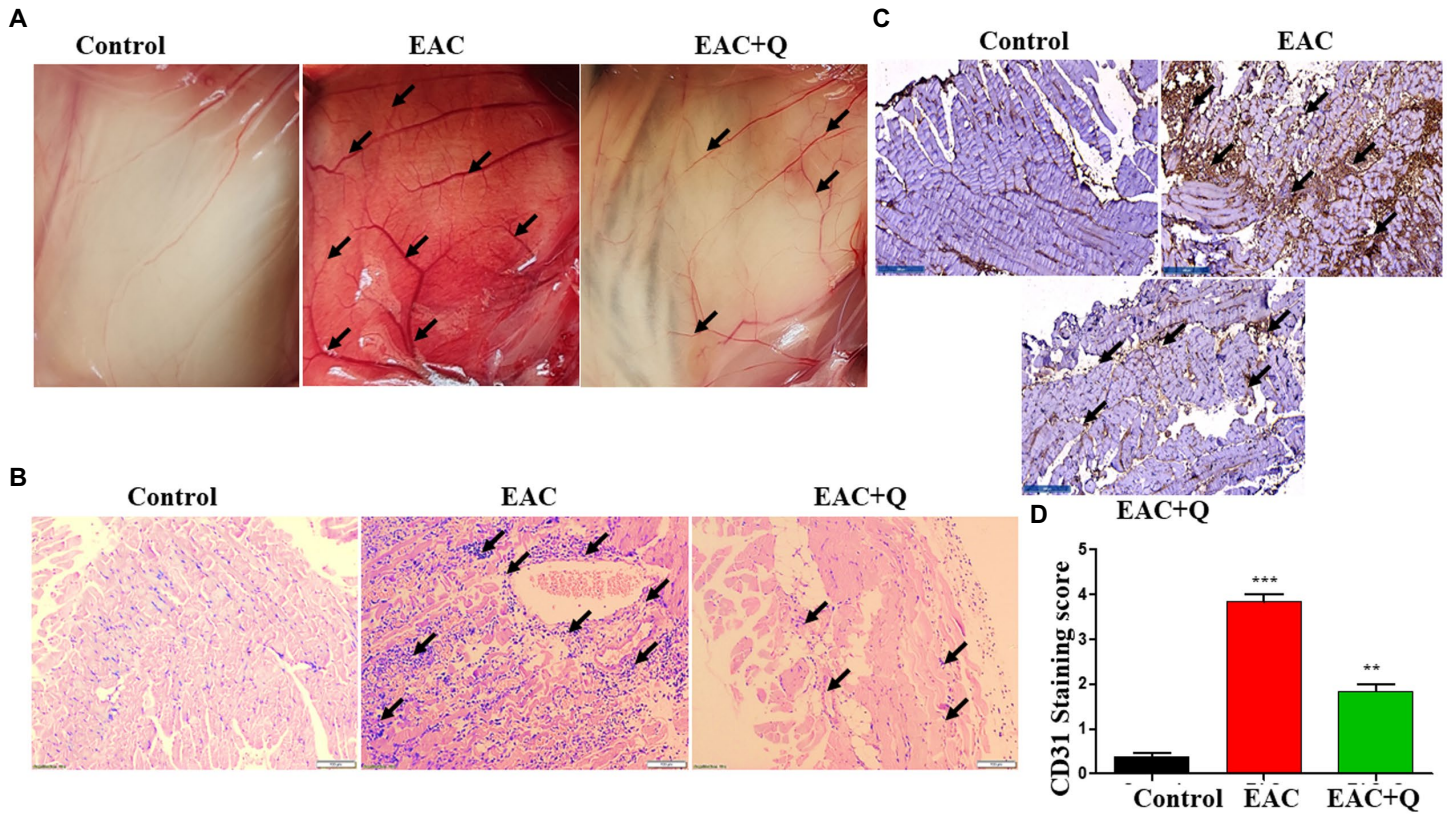
Quercetin suppresses the peritoneal angiogenesis *via* CD31 angiogenic marker. **(A)** Representative image shows the decreased peritoneal neo-angiogenesis in quercetin treated EAC mice when compared to control EAC bearing mice (*n* = 5/group). **(B)** Representative histopathology images of peritoneum, H&E staining shows the suppression of inflammatory cells infiltration in quercetin treated EAC mice when compared to control EAC bearing mice (*n* = 5/group). **(C)** Immunohistochemistry images represent the decreased expression of CD31 marker in quercetin treated EAC mice when compared to EAC bearing mice (*n* = 5/group). **(D)** Bar graph represent the scoring of CD31 marker in respective groups (*n* = 5/group). **, *** represents that *p* values less than 0.005 and 0.0005 respectively.

We noticed a significant reduction in the formation of new blood vessels in the peritoneum of quercetin treated EAC animals as compared to control EAC-bearing animals (Figure 3A). To further validate our findings, we evaluated the inflammatory cells infiltration to the peritoneum. H&E staining was performed and we found a prominent reduction in the levels of inflammatory cell infiltration in the peritoneum of quercetin treated EAC animals as compared to EAC bearing animals (Figure 3B). We also evaluated the expression of CD31 by immunohistochemistry. The result confirmed that, quercetin significantly decreased the CD31 expression against EAC bearing control animals (Figure 3C). The scoring of number of positive cells in respective mice group was also performed (Figure 3D). We observed that quercetin treated EAC animals had fewer CD31 positive cells (*p* < 0.0001) which indicated that quercetin inhibited the endothelial cells proliferation. Therefore, these results collectively demonstrated that quercetin inhibits angiogenesis which is evident through reduced elevation of angiogenic markers.

### 3.4. Quercetin ameliorates Ehrlich ascites tumor cells induced hepatic inflammation and fibrosis

**Figure 4:**
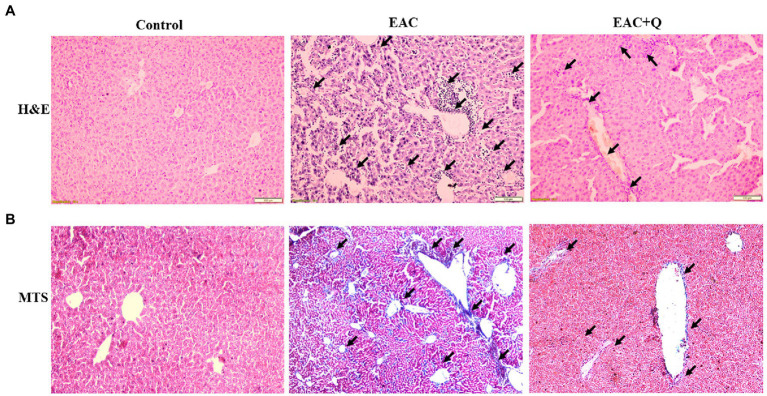
Quercetin ameliorates EAC induced hepatic inflammation and fibrosis. **(A)** Hematoxylin and Eosin staining of quercetin treated EAC animals when compare to EAC bearing animals demonstrating decreased inflammatory cells infiltration (*n* = 5/group). **(B)** Liver section of quercetin treated EAC animals when compared with control EAC bearing animals staining with Trichome Masson’s staining showing decreased liver fibrosis (*n* = 5/group). The arrow mark indicates inflammatory cells infiltration in H&E staining and degree of fibrosis in TMS staining.

To explore the hepatoprotective action of quercetin, we performed H&E staining for all the liver tissue. Quercetin-treated EAC mice liver sections showed significantly decreased inflammation when compared to liver tissues of EAC-bearing control mice (Figure 4A). Further, we conducted Trichome Masson staining (TMS) to assess the liver injury by fibrosis, and we observed that quercetin-treated EAC animals had considerably lower levels of liver fibrosis than EAC-bearing animals (Figure 4B; Supplementary Table S1). Based on these histological data, we confirmed that quercetin protects liver from EAC cells induced inflammation and fibrosis.

### 3.5. Cytotoxicity effect of quercetin and vitamin D3 on breast cancer cell lines

**Figure 5:**
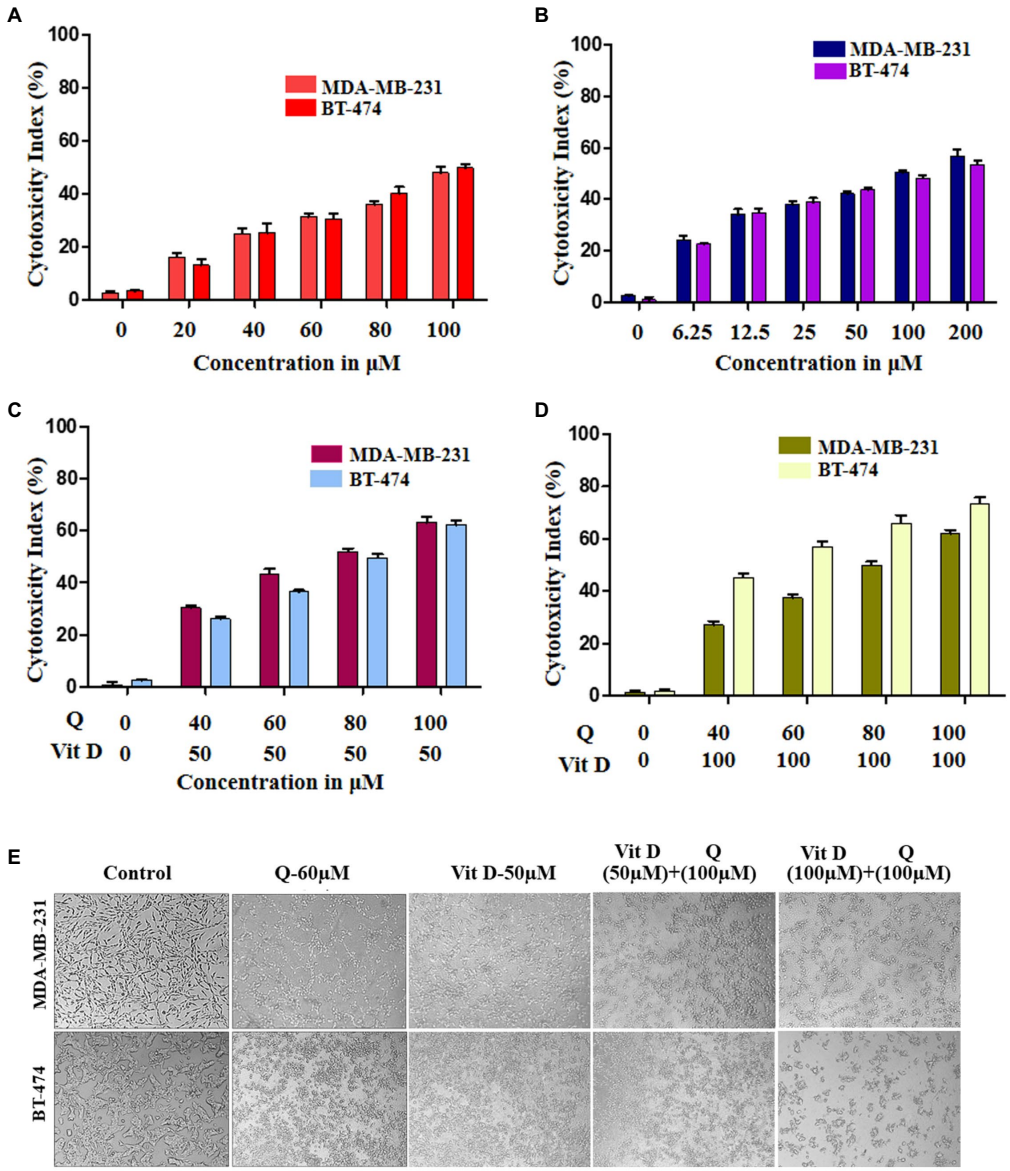
Cytotoxicity effect of quercetin and vitamin D3 on breast cancer cell lines. **(A–D)** Cell death was induced in human breast cancer cell lines (MDA-MB-231 and BT-474) by quercetin, vitamin D3 and combination of both in dose dependent manner for 24 h (*n* = 3). **(E)** Photograph representing the cytotoxicity effect of quercetin, vitamin D3 and combination for 24 h in respective dosage (*n* = 3).

To validate and confirm the vitamin D3 mimicking effect of quercetin, we treated breast cancer cell lines with quercetin (20–100 μM), vitamin D3 (6.25–200 μM), and quercetin (40–100 μM) combined with 50 μM and 100 μM vitamin D3 for 24 h (30). The WST-1 cell proliferation assay results showed that quercetin (*p* < 0.0001) and vitamin D3 (*p* < 0.0001) greatly suppressed the proliferation of human breast cancer cells (Figures 5A,B). Interestingly, we also observed the positive synergetic effect of quercetin and vitamin D3 (*p* < 0.0001) at desired combinations (Figures 5C,D). Our results confirmed that quercetin acts similarly to that of vitamin D3 on breast cancer cells. Also, we calculated the combination index values by using CompuSyn software which indicated the synergetic effects and which was interpreted as shown in Tables 1, 2. From that we observed, quercetin and vitamin D3 had a strong synergetic effect at desired concentrations (Supplementary Figure S1). The photographs were captured under the microscope after 24 h of treatment with the respective concentrations (Figure 5E).

**Table 1:** Interpretation of drug combination index values in MDA-MB-231.

Concentration	Quercetin (μM)			
	40	60	80	100
Vitamin D3 (50 μM)	3.77	1.49	0.88	0.50
Vitamin D3 (100 μM)	2.02	0.98	0.59	0.32

^*^Combination index values < 1 indicate strong synergism.

**Table 2:** Interpretation of drug combination index values in BT-474.

Concentration	Quercetin (μM)			
	40	60	80	100
Vitamin D3 (50 μM)	6.87	2.72	1.16	0.68
Vitamin D3 (100 μM)	1.96	0.89	0.64	0.42

^*^Combination index values < 1 indicate strong synergism.

### 3.6. Quercetin inhibits *ex vivo* angiogenesis

**Figure 6:**
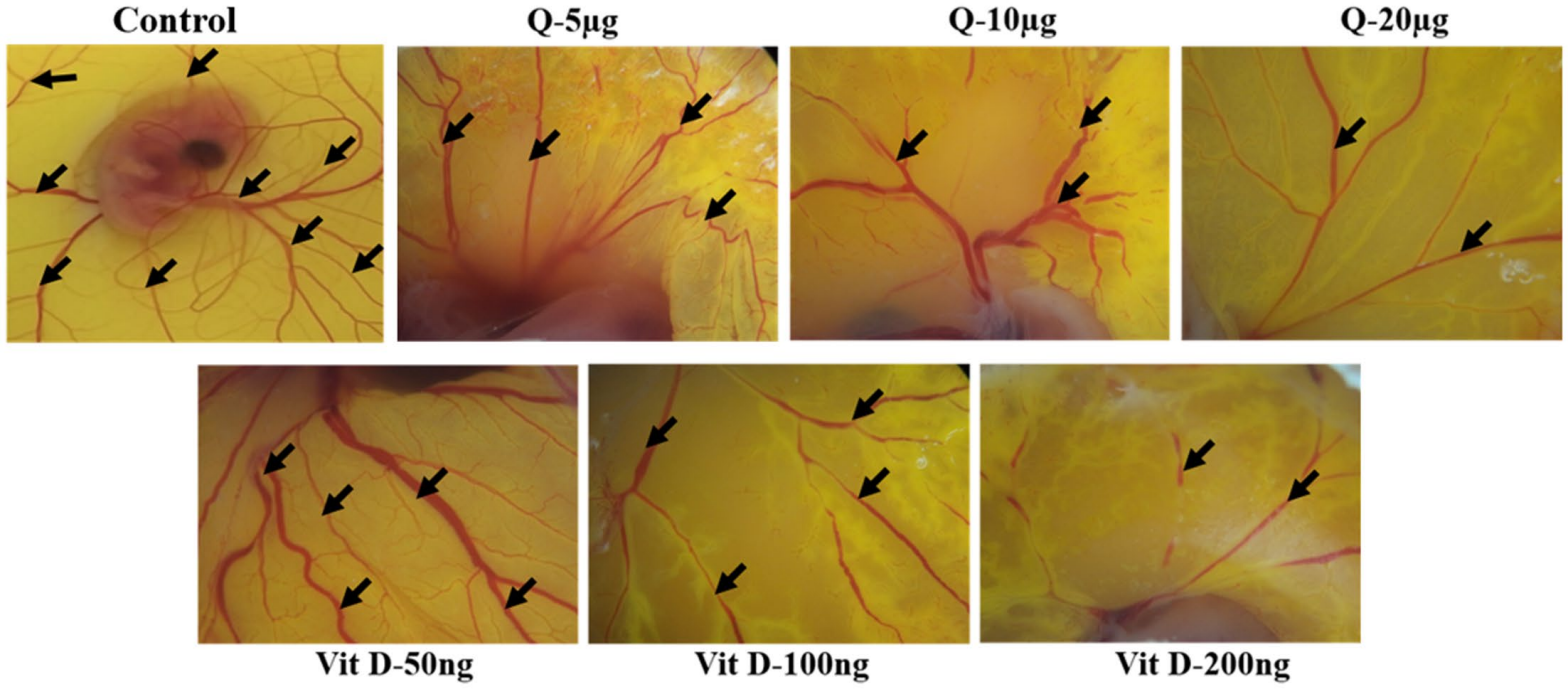
Quercetin inhibits *ex-vivo*-angiogenesis. Anti-angiogenic effect of quercetin and vitamin D3 on chorioallantoic membrane of fertile chicken eggs. Microscopic images were representing the suppression of number of blood vessels in quercetin and vitamin D3 treated eggs compare to control eggs in dose dependent manner (*n* = 5).

Based on our animal experimental evidence and also from *in-vitro* experiments we know that quercetin has anti-tumor activity. To further elucidate the vitamin D mimicking role of quercetin, we performed CAM assay. Our CAM assay data showed that there is a noticeable reduction in the neo-angiogenesis in a dose-dependent manner (Figure 6). Our results also show that, both quercetin and vitamin D3 exhibit a similar inhibitory effect on angiogenesis. Vitamin D3 is a well-established ligand for vitamin D receptor and it acts through this receptor and regulates various gene expression. But whether quercetin interacts with vitamin D receptor is still elusive. Therefore, to confirm these results, further validations are required. Docking studies were performed to know the possible interactions of quercetin as a ligand for vitamin D receptor.

### 3.7. Molecular docking

**Figure 7:**
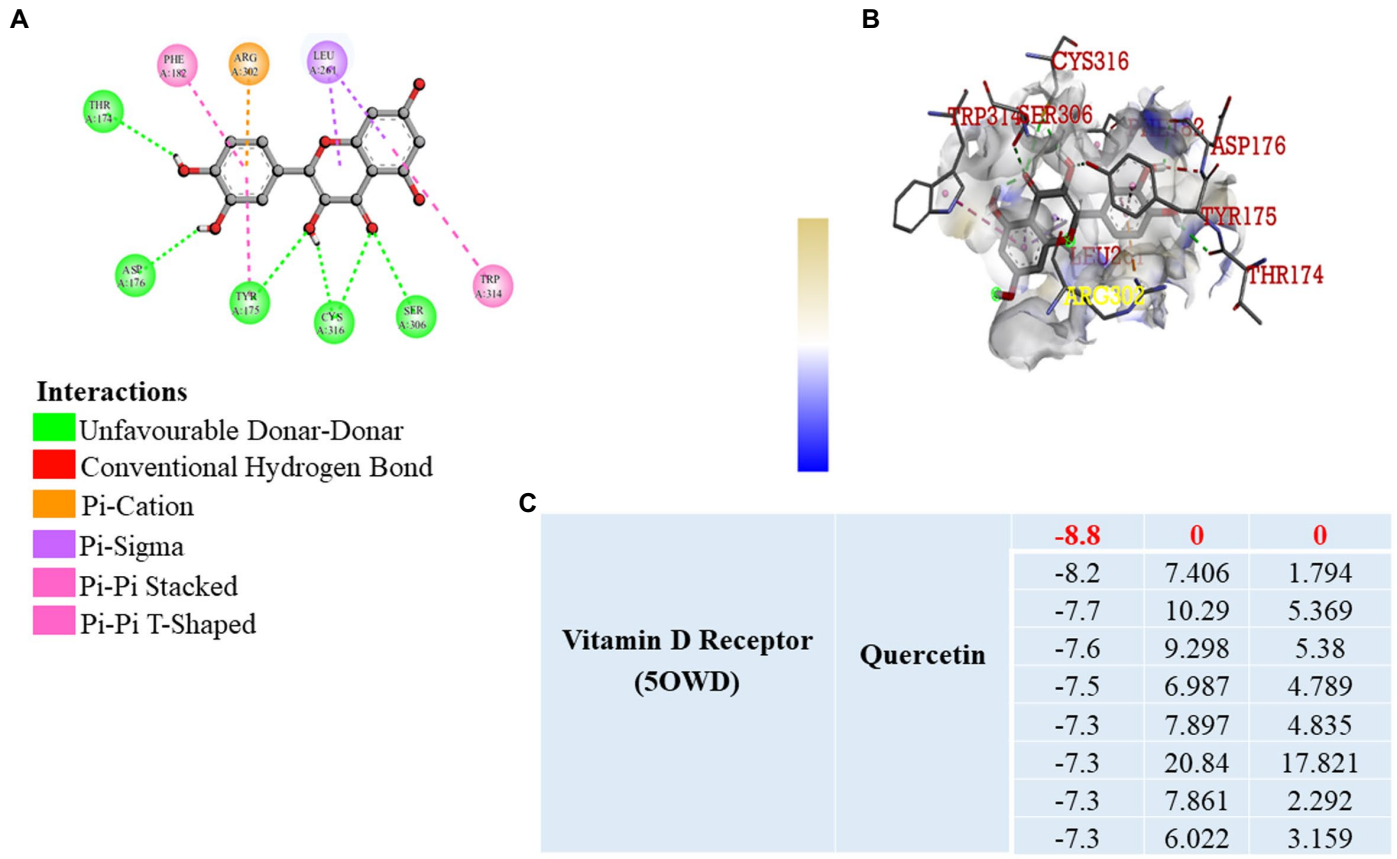
Molecular Docking. **(A,B)** 2D and 3D visualization of protein ligand interaction of vitamin D receptor with quercetin. **(C)** Table represent the binding affinity and RMSD values of the vitamin D receptor docked with quercetin.

Previously published reports suggest that, VDR has a significant role in the prevention of inflammation, fibrosis, and angiogenesis by activating various signaling pathways (31, 32). It is well known that vitamin D3 binds to the VDR, but we wanted to know that whether quercetin interacts with the VDR. As a result, we used software to perform molecular docking to confirm the binding of the quercetin molecule to the vitamin D receptor (Figure 7A). Docking interaction of quercetin as a ligand revealed a higher binding affinity to vitamin D receptor with THR174, ARG 302, ASP 176, TYR 175, CYS 316, and SER 306 as the amino acids that bind to quercetin ligand based on affinity scoring and binding posture (Figures 7B,C). Our study focused primarily on the interaction of quercetin with VDR and we did not examine the interaction of other flavonoids with VDR. As per the data of docking study, we confirmed that quercetin has a high affinity for VDR and may be involved in the prevention of angiogenesis, inflammation, and fibrosis by activating signaling pathways.

## 4. Discussion

The number of deaths in patients with breast cancer is increasing day by day. Many breast cancer patients frequently develop comorbidities associated complications, which raises the total mortality rate. Our recent study linked the breast cancer associated angiogenesis with liver inflammation and fibrosis (9). However, the molecular mechanism underlying the breast cancer associated liver inflammation and fibrosis is still not fully understood. Therefore, we further extrapolated our study to address the knowledge in gap and also potentially develop the therapeutic strategy to inhibit the breast cancer and associated tumor angiogenesis to prevent the liver inflammation and fibrosis.

**Figure 8:**
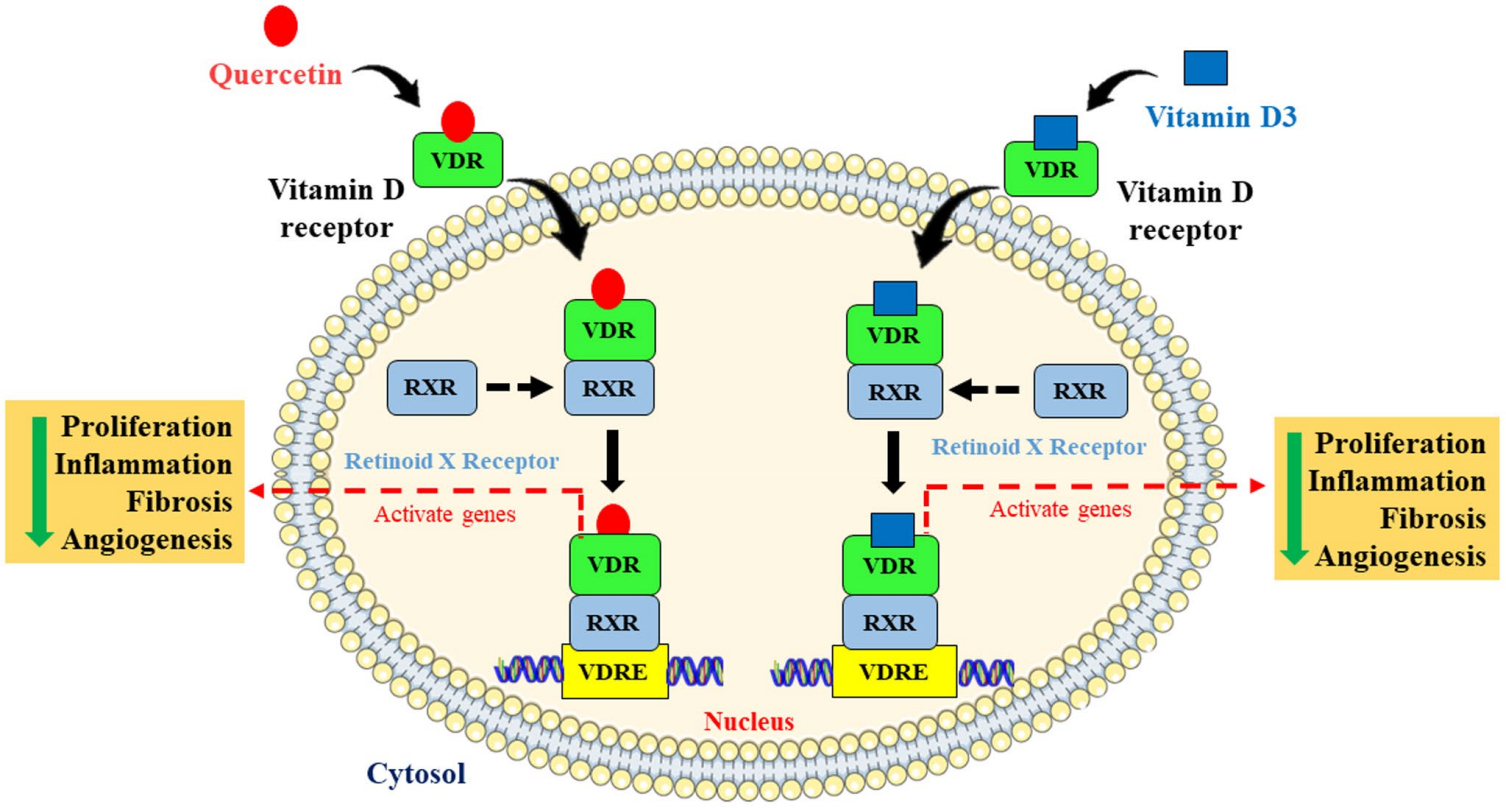
Schematic representation and molecular mechanism of quercetin mimicking the role of vitamin D *via* vitamin D receptor. Quercetin mimics vitamin D through activating the vitamin D receptor by forming dimers with retinoid X receptor and is involved in the inhibition of cell proliferation, angiogenesis, inflammation, and fibrosis *via* activating Vitamin D response elements (VDRE).

Understanding the link between breast cancer and hepatic dysfunction may help to reduce the number of cancer-related fatalities not only in breast cancer patients but also in other cancer patients too. Therefore, in this novel and innovative study, we explored the most possible action of quercetin on breast cancer-induced liver inflammation and fibrosis *via* VDR (Figure 8).

Natural compounds have a wide range of beneficial properties, making them promising anti-cancer compounds. Here, we demonstrated the new role of quercetin in which it inhibited breast cancer-related liver injury. In this model, as the tumor cell number increases, the body weight rapidly increases, and as a result of body weight and tumor burden, the mortality rate also rises. Compelling pieces of evidence showed an association between weight gain and breast cancer mortality. Our findings suggest the potential efficacy of quercetin in weight control of animals with breast cancer. Future clinical trials are warranted to investigate if quercetin treatment could improve the weight control and survival of breast cancer patients (33). Therefore, reduction in the body weight significantly helps to reduce the tumor burden as well as mortality rate in our mouse model. In this study, we found that the body weight of the quercetin treated EAC animals was considerably decreased than that of the positive control group. Ascites fluid is a direct source of various secretary growth factors and cytokines and these molecules play a major role in cancer-associated comorbidities and also mortality increases from such complications. Therefore, it is necessary to reduce the secretion of ascites fluid. Also, ascites volume increases with the tumor cell proliferation and stagewise progression of tumor growth (34). Quercetin-treated EAC animals significantly had much less ascites fluid than untreated control EAC-bearing animals. Along with the decreased ascites volume, we also found reduced viability of EAC cells in the quercetin-treated group. Importantly, our findings showed that quercetin has a hepatoprotective role and it aids in physiological liver function. Increased liver weight and liver enzyme activity are the major hallmarks of liver inflammation and fibrosis (35). When we observed the overall liver weight of quercetin treated and untreated EAC animals, we found that quercetin treated mice livers had much lower liver weight than that of EAC-bearing animals. Furthermore, we found decreased levels of liver function associated enzymes such as ALP, AST, and ALT in quercetin treated EAC mice compared to control EAC mice. These experimental pieces of evidence strongly confirmed that quercetin has a positive effect on maintaining liver function.

Both experimental and clinical studies have demonstrated tumor angiogenesis plays a prominent role in cancer-related diseases (36, 37). The increased mortality rate of cancer-related deaths is directly linked to tumor angiogenesis, which pumps angiogenic factors and inflammatory cytokines toward normal cells triggering them to divide faster (38). Further, our experimental observational suggests that peritoneal angiogenesis is significantly reduced in quercetin-treated EAC animals compared to control EAC-bearing animals. Furthermore, we examined the level of inflammatory cell infiltration in the peritoneum of quercetin-treated EAC mice as well as control mice. Our immunohistochemistry data showed that quercetin-treated EAC animals had less immune cell infiltration than control EAC-bearing animals. The expression of well-established angiogenesis marker, CD31 level was also found to be decreased in quercetin treated peritoneum when compared to control peritoneum (39). This revealed that CD31 positive endothelial cell proliferation may be inhibited by quercetin and which in turn leads to decreased tumor angiogenesis in treated mice compared to control mice. Overall, these findings revealed the potential novel role of quercetin in the prevention of hepatic inflammation and fibrosis.

Cancer cells secrete a high level of inflammatory and angiogenic factors, these factors are key mediators of liver inflammation and fibrosis (40). Our H&E and TMS staining data of liver tissue also showed decreased hepatic inflammation and fibrosis in quercetin treated EAC mice when compared to control mice. These findings strongly suggest that quercetin treated EAC animals had a lower level of inflammatory cell infiltration and fibrosis.

When we were searching for a potential target of quercetin, we came across few interesting articles which predicted that the Vitamin D receptor acts as a possible target of quercetin (23, 41). They also indicated that quercetin may serve as a potent ligand for Vitamin D receptors and may activate its function. Activation of VDR by its ligand is involved in the inhibition of tumor cells proliferation, invasion, migration, and angiogenesis (42, 43). Some of these studies strongly indicate that lack of vitamin D increases the rate and risk of breast cancer (44, 45). Vitamin D3 ameliorates the probability of breast cancer by inhibiting the hallmarks of cancer such as cell proliferation, invasion, and metastasis (46). To explore the further possible mechanism of ameliorating effect of quercetin on EAC-induced liver inflammation we treated breast cancer cells with vitamin D3 in a dose-dependent manner with quercetin. Quercetin and vitamin D3 both significantly suppressed the proliferation of human breast cancer cell lines (MDA-MB-231 and BT-474) in a similar fashion. We also noticed that the combination of quercetin and vitamin D3 shows a strong synergetic effect by inhibiting the breast cancer cells proliferation. Although some research studies have delved into the effects of vitamin D3 on angiogenesis, which is one of the most critical components, it may permit metastasis. Our CAM assay results also showed that both quercetin and vitamin D3 inhibited neo-angiogenesis. These results strongly suggest that quercetin appears to mimic the action of vitamin D3 *via* VDR. Interestingly, one or two studies reported that quercetin can act as a ligand for Vitamin D receptor (VDR) which further may activate and regulate VDR-mediated signaling pathways (22, 23). A recent study showed that VDR acts as a possible therapeutic target for breast cancer (41). Some of these studies also suggested that VDR activation helps in the prevention of hepatic fibrosis in chronic liver injury (47, 48). To confirm the interaction between quercetin and VDR, we conducted a molecular docking study and confirmed the interaction between quercetin and VDR. According to our molecular docking results, quercetin has a strong binding affinity for VDR with an excellent affinity score and binding posture. Both quercetin and vitamin D3 are structurally distinct. However, the functional similarity between the quercetin and vitamin D3 shares similar hydrogen bonding networks between the hydroxyl groups of the six membered ring of the ligand and also interacts with similar amino acid residues while interacting with VDR (23). Vitamin D receptor is very commonly associated with the retinoic acid receptor (RAR) or retinoid X receptor (RXR) (49). Upon dimerization, the VDR-RXR heterodimer binds to vitamin D-responsive elements and activates specific target genes expression for the physiological process (50). To our knowledge, no reports have shown the direct interaction of Quercetin with RAR/RXR. This predicts the possible potential role of quercetin and its action which may regulate various gene expression *via* activating VDR and making it to bind Vitamin D response elements (VDRE).

We were the first to report the potent interaction between quercetin and VDR, as well as an ameliorating effect of quercetin on breast cancer-induced hepatic inflammation and fibrosis. Limitation of our study is that we did not investigate the role of liver-specific vitamin D target genes in the prevention of breast cancer induced liver injury. Here, we tried to figure out the molecular mechanism and ameliorating effect of quercetin on breast cancer-induced liver inflammation and fibrosis *via* VDR and in near future we will further extend our study to validate the effect of quercetin on vitamin D target genes using breast cancer cells and liver specific cell lines.

## 5. Conclusion

Through these findings, we explored the novel role of quercetin and its ameliorating effect on breast cancer-induced liver injury. Quercetin inhibited the elevation of liver enzymes, hepatic inflammation, and fibrosis in EAC-induced animals, proving its strong hepatoprotective effect. Furthermore, our findings strongly suggest that quercetin mimics vitamin D3 and our molecular docking analysis demonstrated that quercetin strongly binds to VDR. It is already known that activation of this receptor suppresses hepatic inflammation and fibrosis. Therefore, our findings in animal model clearly show that quercetin can suppress breast cancer-induced liver inflammation and fibrosis. The mechanism is possibly through the activation of VDR. Future clinical trials are warranted to investigate the effect of quercetin on liver inflammation and fibrosis in breast cancer patients and the potential to reduce the co-morbidity and improve cancer survival. In our future studies we will try to explore the role of some of the direct vitamin D target genes in mediating quercetin effects and we will also demonstrate the complete molecular mechanism behind quercetin mediated VDR activation and its therapeutic impact on breast cancer induced hepatic inflammation and fibrosis.

## Data availability statement

The datasets presented in this study can be found in online repositories. The names of the repository/repositories and accession number(s) can be found in the article/Supplementary material.

## Ethics statement

The animal study was reviewed and approved by JSS AHER Institutional Ethical Committee.

## Author contributions

NS performed the experiments, analyzed the data, and drafted the manuscript. VS, LP, SD, and CB helped during *in vivo* and *in vitro* experiments. AS provided intellectual inputs to the manuscript. SB and SP aided in docking studies. SM and RV provided intellectual inputs to the manuscript. PS designed the project, provided overall supervision and intellectual guidance, and drafted the manuscript. All authors contributed to the article and approved the submitted version.

## Funding

This work was supported by Department of Science and Technology funding through the “Promotion of University Research and Scientific Excellence” (PURSE) scheme with sanction number: SR/PURSE/2021/81 (G). NS acknowledges JSS AHER (for salary support number: REG/DIR(R)/JSSURF/29(2)/202-21) and KSTA (grant number: K.S.T.A/K/30/202-21). PS and VS (for stipend support) acknowledges Ramalingaswami Re-entry fellowship, Department of Biotechnology (DBT), Govt. of India. We also would like to acknowledge the infrastructure support provided by DST-FIST to the Center of Excellence in Molecular Biology and Regenerative Medicine (CEMR) Laboratory (CR-FST-LS-1/2018/178) and the Department of Biochemistry (SR/FST/LS-1-539/2012). SM and SD would like to thank the DBT for grant support (N0-BT/PR29598/PFN/20/1392/2018) and Senior Research Fellowship. SM would like to thank the Special Interest Group in Cancer Biology and Cancer Stem Cells (SIG-CBCSC).

## Conflict of interest

The authors declare that the research was conducted in the absence of any commercial or financial relationships that could be construed as a potential conflict of interest.

## Publisher’s note

All claims expressed in this article are solely those of the authors and do not necessarily represent those of their affiliated organizations, or those of the publisher, the editors and the reviewers. Any product that may be evaluated in this article, or claim that may be made by its manufacturer, is not guaranteed or endorsed by the publisher.

## Supplementary material

SUPPLEMENTARY FIGURE S1 Graph indicate the synergetic effects of quercetin and vitamin D3.

SUPPLEMENTARY TABLE S1 The table shows the scoring of the pathological changes of H&E and MTS staining. 3-4 score indicates severe, 1-2 score indicates moderate and 0 indicates none.

## References

[1] Sung H Ferlay J Siegel RL Laversanne M Soerjomataram I Jemal A. Global cancer statistics 2020: GLOBOCAN estimates of incidence and mortality worldwide for 36 cancers in 185 countries. CA Cancer J Clin. (2021) 71:209–49. doi: 10.3322/caac.21660, PMID: 33538338

[2] Momenimovahed Z Salehiniya H. Incidence, mortality and risk factors of cervical ancer in the world. Biomed Res Ther. (2017) 4:1795–11. doi: 10.15419/bmrat.v4i12.386

[3] Nazario HE Lepe R Trotter JF. Metastatic breast cancer presenting as acute liver failure. Gastroenterol Hepatol. (2011) 7:65–6. PMID: 21346858 PMC3038322

[4] Fu MR Axelrod D Guth AA Cleland CM Ryan CE Weaver KR. Comorbidities and quality of life among breast cancer survivors: a prospective study. J Pers Med. (2015) 5:229–42. doi: 10.3390/jpm5030229, PMID: 26132751 PMC4600145

[5] Lorincz AM Sukumar S. Molecular links between obesity and breast cancer. Endocr Relat Cancer. (2006) 13:279–92. doi: 10.1677/erc.1.00729 16728564

[6] Lee YS Lee HS Chang SW Lee CU Kim JS Jung YK. Underlying nonalcoholic fatty liver disease is a significant factor for breast cancer recurrence after curative surgery. Medicine. (2019) 98:e17277. doi: 10.1097/MD.0000000000017277, PMID: 31574842 PMC6775430

[7] Rashid NS Grible JM Clevenger CV Harrell JC. Breast cancer liver metastasis: current and future treatment approaches. Clin Exp Metastasis. (2021) 38:263–77. doi: 10.1007/s10585-021-10080-4, PMID: 33675501 PMC8211035

[8] Xu Z Yuan JJ Jiang J Ma L. Liver metastasis occurring within four months of early breast cancer diagnosis: a case report and literature review. Case Rep Oncol. (2022) 15:827–32. doi: 10.1159/000526029, PMID: 36825109 PMC9941772

[9] Sannappa Gowda NG Shiragannavar VD Prabhuswamimath SC Tuladhar S Chidambaram SB Santhekadur PK. Ehrlich ascites carcinoma mice model for studying liver inflammation and fibrosis. Adv Cancer Biol Metastasis. (2022) 100029. doi: 10.1016/j.adcanc.2022.100029

[10] Trefts E Gannon M Wasserman DH. The liver. Curr Biol. (2017) 27:1147–51. 10.1016/j.cub.2017.09.019 PMC5897118 29112863

[11] Zuazo-Gaztelu I Casanovas O. Unraveling the role of angiogenesis in cancer ecosystems. Front Oncol. (2018) 8:248. doi: 10.3389/fonc.2018.00248, PMID: 30013950 PMC6036108

[12] Li H. Angiogenesis in the progression from liver fibrosis to cirrhosis and hepatocelluar carcinoma. Expert Rev Gastroenterol Hepatol. (2021) 15:217–33. doi: 10.1080/17474124.2021.1842732, PMID: 33131349

[13] Sugino T Yamaguchi T Hoshi N Kusakabe T Ogura G Goodison S. Sinusoidal tumor angiogenesis is a key component in hepatocellular carcinoma metastasis. Clin Exp Metastasis. (2008) 25:835–41. doi: 10.1007/s10585-008-9199-6, PMID: 18712609 PMC3431607

[14] Zhang L Wang JN Tang JM Kong X Yang JY Zheng F. VEGF is essential for the growth and migration of human hepatocellular carcinoma cells. Mol Biol Rep. (2012) 39:5085–93. doi: 10.1007/s11033-011-1304-2, PMID: 22161247 PMC4354771

[15] Yang W Li Z Qin R Wang X An H Wang Y. YY1 promotes endothelial cell-dependent tumor angiogenesis in hepatocellular carcinoma by transcriptionally activating VEGFA. Front Oncol. (2019) 9:1187. doi: 10.3389/fonc.2019.01187, PMID: 31799179 PMC6868052

[16] Mossenta M Busato D Baboci L Cintio FD Toffoli G Bo MD. New insight into therapies targeting angiogenesis in hepatocellular carcinoma. Cancers. (2019) 11:1086. doi: 10.3390/cancers11081086, PMID: 31370258 PMC6721310

[17] Pan X Ma X Jiang Y Wen J Yang L Chen D. A comprehensive review of natural products against liver fibrosis: flavonoids, Quinones, Lignans, phenols, and acids. Evid Based Complement Alternat Med. (2020) 2020:7171498. doi: 10.1155/2020/7171498 33082829 PMC7556091

[18] Tang SM Deng XT Zhou J Li QP Ge XX Miao L. Pharmacological basis and new insights of quercetin action in respect to its anti-cancer effects. Biomed Pharmacother. (2020) 121:109604. doi: 10.1016/j.biopha.2019.109604, PMID: 31733570

[19] Qiu D Yan X Xiao X Zhang G Wang Y Cao J. To explore immune synergistic function of Quercetin in inhibiting breast cancer cells. Cancer Cell Int. (2021) 21:632. doi: 10.1186/s12935-021-02345-5, PMID: 34838003 PMC8626953

[20] Hashemzaei M Delarami Far A Yari A Heravi RE Tabrizian K Taghdisi SM. Anticancer and apoptosis-inducing effects of quercetin in vitro and in vivo. Oncol Rep. (2017) 21:819–28. 10.3892/or.2017.5766 PMC5561933 28677813

[21] Kato S. The function of vitamin D receptor in vitamin D action. J Biochem. (2000) 127:717–22. doi: 10.1093/oxfordjournals.jbchem.a022662 10788778

[22] Lee KY Choi HS Choi HS Chung KY Lee BJ Maeng HJ. Quercetin directly interacts with vitamin D receptor (VDR): structural implication of VDR activation by Quercetin. Biomol Ther. (2016) 24:191–8. doi: 10.4062/biomolther.2015.122, PMID: 26902087 PMC4774501

[23] Inoue J Choi JM Yoshidomi T Yashiro T Sato R. Quercetin enhances VDR activity, leading to stimulation of its target gene expression in Caco-2 cells. J Nutr Sci Vitaminol (Tokyo). (2010) 56:326–30. doi: 10.3177/jnsv.56.326, PMID: 21228504

[24] Elkhawaga OA Gebril S Salah N. Evaluation of anti-tumor activity of metformin against Ehrlich ascites carcinoma in Swiss albino mice. Egypt J Basic Appl Sci. (2016) 6:116–23. doi: 10.1080/2314808X.2019.1676003

[25] Oršolić N Odeh D Jembrek MJ Knežević J Kučan D. Interactions between Cisplatin and Quercetin at physiological and Hyperthermic conditions on cancer cells in vitro and in vivo. Molecules. (2020) 25:3271. doi: 10.3390/molecules25143271, PMID: 32709143 PMC7397216

[26] Muta K Nakazawa Y Obata Y Inoue H Torigoe K Nakazawa M. An inhibitor of Krüppel-like factor 5 suppresses peritoneal fibrosis in mice. Perit Dial Int. (2021) 41:394–403. doi: 10.1177/0896860820981322, PMID: 33522431

[27] Prasanna KS Shilpa P Salimath BP. Withaferin a suppresses the expression of vascular endothelial growth factor in Ehrlich ascites tumor cells via Sp1 transcription factor. Curr Trends Biotechnol Pharm. (2009) 3:138–48. doi: 10.3892/or.2017.5766

[28] Akinwunmi OA John OO Habib T Segun OL Cecilia OB Mobolaji TA. Molecular docking analysis of apigenin and quercetin from ethylacetate fraction of Adansonia digitata with malaria-associated calcium transport protein: An in silico approach. Heliyon. (2019) 5:e02248. doi: 10.1016/j.heliyon.2019.e02248 31687530 PMC6819832

[29] Tajiri K Shimizu Y. Liver physiology and liver diseases in the elderly. World J Gastroenterol. (2013) 19:8459–67. doi: 10.3748/wjg.v19.i46.8459, PMID: 24379563 PMC3870491

[30] Tekin G Unlu A Ozturk B. Effects of Quercetin and vitamin D on MCF-7 cell proliferation with real-time cell analysis. Int J Sci Res. (2017) 6:708–12. doi: 10.21275/ART20171341

[31] Yazdani S Poosti F Toro L Wedel J Mencke R Mirković K. Vitamin D inhibits lymphangiogenesis through VDR-dependent mechanisms. Sci Rep. (2017) 7:44403. doi: 10.1038/srep44403, PMID: 28303937 PMC5355885

[32] Donate-Correa J Domínguez-Pimentel V Méndez-Pérez ML Muros-de-Fuentes M Mora-Fernández C Martín-Núñez E. Selective vitamin D receptor activation as anti-inflammatory target in chronic kidney disease. Mediat Inflamm. (2014) 670475, 1543–1551. doi: 10.1155/2014/670475 PMC3913352 24511210

[33] Jung AY Hüsing A Behrens S Krzykalla J Obi N Becher H. Postdiagnosis weight change is associated with poorer survival in breast cancer survivors: a prospective population-based patient cohort study. Int J Cancer. (2021) 148:18–27. doi: 10.1002/ijc.33181 32621760

[34] Mingtian W Tinghan Y Xiangzheng C Yangping W Xiangbing D Wanbin H. Malignant ascites-derived exosomes promote proliferation and induce carcinoma-associated fibroblasts transition in peritoneal mesothelial cells. Oncotarget. (2017) 8:42262–71. doi: 10.18632/oncotarget.15040 28178689 PMC5522065

[35] Ma J Liu W Yan X Wang Q Zhao Q Xue Y. Inhibition of endothelial cell proliferation and tumor angiogenesis by up-regulating NDRG2 expression in breast cancer cells. PLoS One. (2012) 7:e32368. doi: 10.1371/journal.pone.0032368 22393400 PMC3290656

[36] Tousson E Hafez E Abo Gazia MM Salem SB Mutar TF. Hepatic ameliorative role of vitamin B17 against Ehrlich ascites carcinoma-induced liver toxicity. Environ Sci Pollut Res Int. (2020) 27:9236–46. doi: 10.1007/s11356-019-06528-6, PMID: 31916166

[37] Dore-Savard L Lee E Kakkad S Popel AS Bhujwalla ZM. The Angiogenic Secretome in VEGF overexpressing breast cancer Xenografts. Sci Rep. (2016) 6:39460. doi: 10.1038/srep39460, PMID: 27995973 PMC5171865

[38] Colpaert CG Vermeulen PB Benoy I Soubry A van Roy F van Beest P. Inflammatory breast cancer shows angiogenesis with high endothelial proliferation rate and strong E-cadherin expression. Br J Cancer. (2003) 88:718–25. doi: 10.1038/sj.bjc.6600807, PMID: 12618881 PMC2376338

[39] Belakavadi M Salimath BP. Mechanism of inhibition of ascites tumor growth in mice by curcumin is mediated by NF-kB and caspase activated DNase. Mol Cell Biochem. (2005) 273:57–67. doi: 10.1007/s11010-005-7717-2, PMID: 16013440

[40] Nitin S Deepak B Jagadish PR Pankaj BP Savita ST Veena BP. Inflammation and cancer. Ann Afr Med. (2019) 18:121–6. 31417011 10.4103/aam.aam_56_18 PMC6704802

[41] Murray A Madden SF Synnott NC Klinger R O'Connor D O'Donovan N. Vitamin D receptor as a target for breast cancer therapy. Endocr Relat Cancer. (2017) 24:181–95. doi: 10.1530/ERC-16-0463, PMID: 28213567

[42] Randall LM Monk BJ Darcy KM Tian C Burger RA Liao SY. Markers of angiogenesis in high-risk, early-stage cervical cancer: a gynecologic oncology group study. Gynecol Oncol. (2009) 112:583–9. doi: 10.1016/j.ygyno.2008.11.013, PMID: 19110305 PMC2858218

[43] Zheng Y Trivedi T Lin RC Fong-Yee C Nolte R Manibo J. Loss of the vitamin D receptor in human breast and prostate cancers strongly induces cell apoptosis through downregulation of Wnt/β-catenin signaling. Bone Res. (2017) 5:17023. doi: 10.1038/boneres.2017.23, PMID: 28944088 PMC5605769

[44] Williams JD Aggarwal A Swami S Krishnan AV Ji L Albertelli MA. Tumor autonomous effects of vitamin D deficiency promote breast cancer metastasis. Endocrinology. (2016) 157:1341–7. doi: 10.1210/en.2015-2036, PMID: 26934299 PMC4816742

[45] Li Z Wu L Zhang J Huang X Thabane L Li G. Effect of vitamin D supplementation on risk of breast cancer: a systematic review and meta-analysis of randomized controlled trials. Front Nutr. (2021) 8:655727. doi: 10.3389/fnut.2021.655727, PMID: 33869269 PMC8049142

[46] Feldman D Krishnan AV Swami S Giovannucci E Feldman BJ. The role of vitamin D in reducing cancer risk and progression. Nat Rev Cancer. (2014) 14:342–57. doi: 10.1038/nrc3691, PMID: 24705652

[47] Zhang R Wang M Wang M Zhang L Ding Y Tang Z. Vitamin D level and vitamin D receptor genetic variation were involved in the risk of non-alcoholic fatty liver disease: a case-control study. Front Endocrinol. (2021) 6:648844. doi: 10.3389/fendo.2021.648844 PMC8377425 34421816

[48] Sun S Xu M Zhuang P Chen G Dong K Dong R. Effect and mechanism of vitamin D activation disorder on liver fibrosis in biliary atresia. Sci Rep. (2021) 11:19883. doi: 10.1038/s41598-021-99158-3, PMID: 34615940 PMC8494743

[49] Jimenez-Lara AM Aranda A. Interaction of vitamin D and retinoid receptors on regulation of gene expression. Horm Res. (2000) 54:301–5. doi: 10.1159/000053276 11595822

[50] Wan LY Zhang YQ Chen MD Liu CB Wu JF. Relationship of structure and function of DNA-binding domain in vitamin D receptor. Molecules. (2015) 20:12389–99. doi: 10.3390/molecules200712389, PMID: 26198224 PMC6332450

