# Supplementary figures and images for: Quercetin activates vitamin D receptor and ameliorates breast cancer induced hepatic inflammation and fibrosis
Source: Front Nutr. 2023 Apr 20;10:1158633. doi: 10.3389/fnut.2023.1158633 (PMC10157213; doi:10.3389/fnut.2023.1158633)

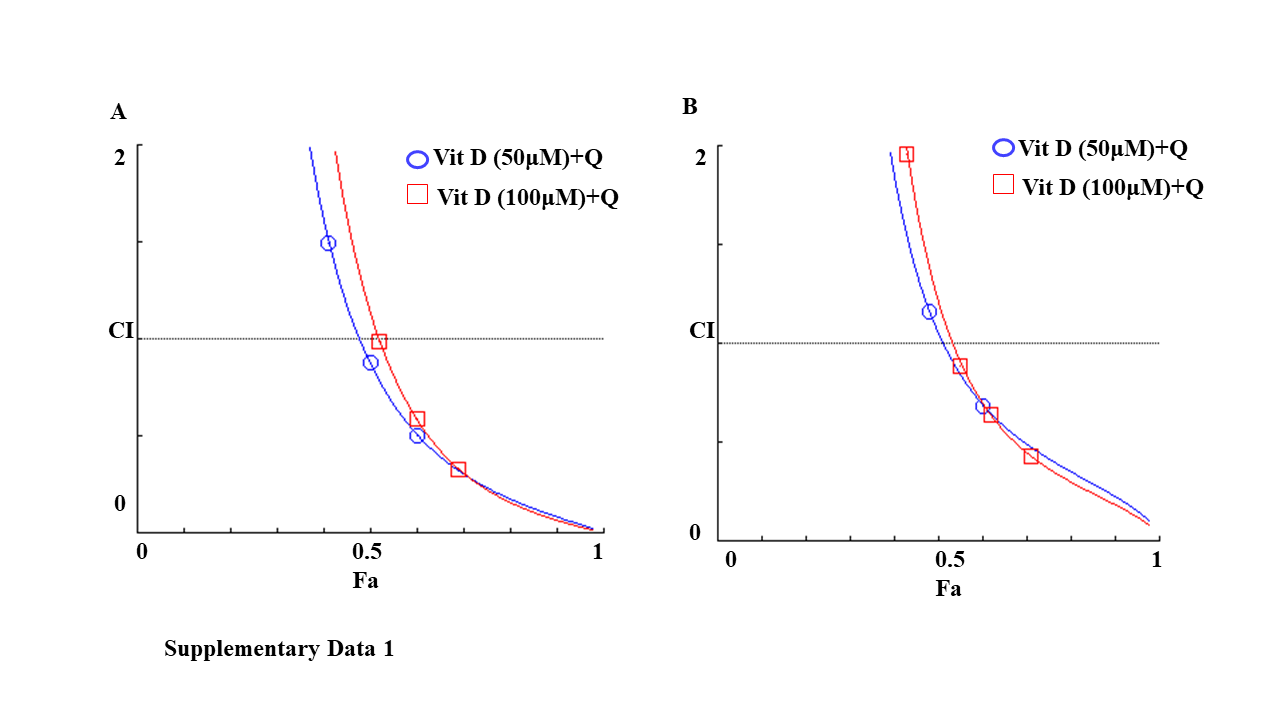

Supplement: SUPPLEMENTARY FIGURE S1 — Graph indicate the synergetic effects of quercetin and vitamin D3. [file Image_1.TIF]

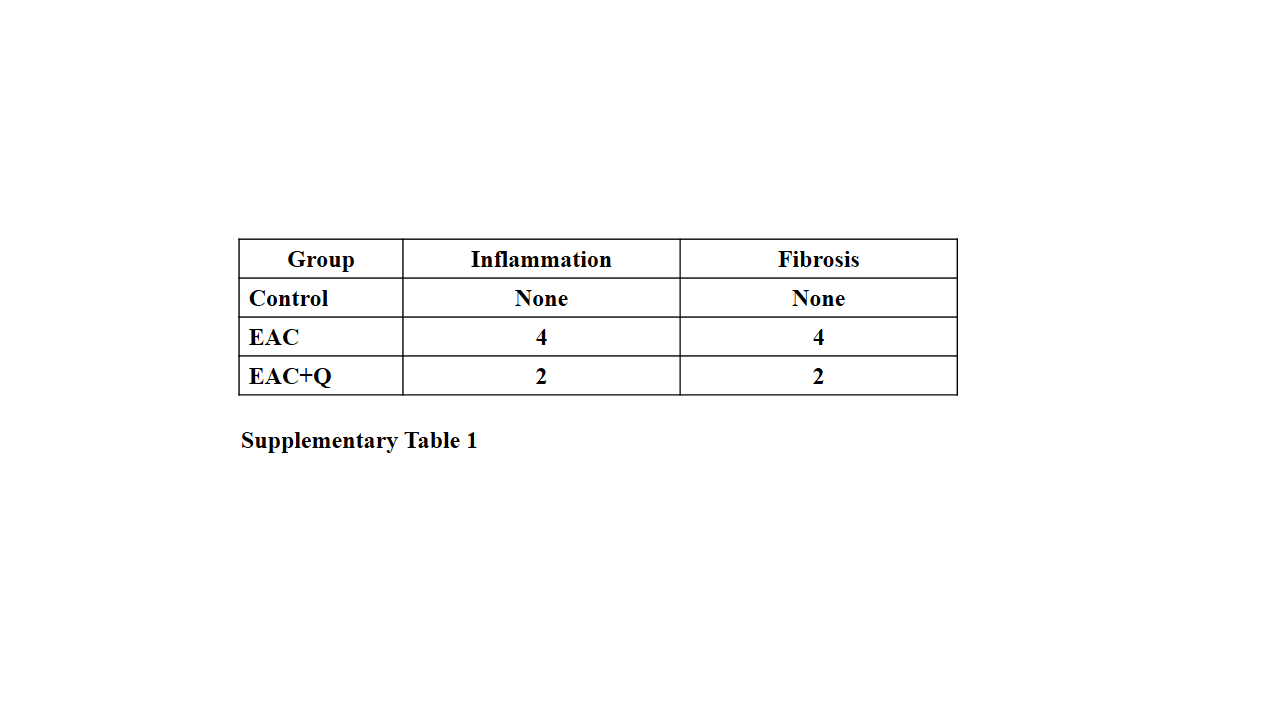

Supplement: SUPPLEMENTARY TABLE S1 — The table shows the scoring of the pathological changes of H&E and MTS staining. 3-4 score indicates severe, 1-2 score indicates moderate and 0 indicates none. [file Image_2.TIF]
